# Histological transformation in lung cancer: a single-arm meta-analysis and systematic review

**DOI:** 10.1186/s12885-026-16114-y

**Published:** 2026-05-21

**Authors:** Lefei Hu, Xunxia Zhu, Xiaoyu Chen, Fuzhi Yang, Shuai Jiang, Shixiang Guo, Mingfeng Wei, Zheng Li, Xiaoyong Shen

**Affiliations:** Department of Thoracic Surgery, Huadong Hospital, 221# West Yanan Road, Shanghai, 200040 China

**Keywords:** Lung cancer, Histological transformation, EGFR-TKIs

## Abstract

**Background:**

Histological transformation represents an important mechanism of acquired resistance to epidermal growth factor receptor tyrosine kinase inhibitors (EGFR-TKIs) in EGFR-mutant lung cancer; however, its incidence, timing, and post-transformation outcomes remain incompletely characterized.

**Methods:**

We conducted a systematic review and meta-analysis in accordance with PRISMA guidelines and Cochrane recommendations. PubMed, Web of Science and Embase were searched up to November 2025 for studies reporting histological transformation in EGFR-mutant lung cancer following EGFR-TKI resistance. Eligible studies included prospective or retrospective studies and consecutive case series (sample size ≥ 10 or ≥3events). Random-effects models were applied to pool transformation proportions and survival outcomes, and Kaplan–Meier–based methods were used to synthesize time-to-event data.

**Results:**

A total of 35 studies were included. Among patients with non–small cell lung cancer (NSCLC) who developed resistance to EGFR-TKIs, the pooled proportion of transformation to high-grade neuroendocrine carcinoma (HGNEC) was 6% (95% CI, 5%–8%), while transformation to non-neuroendocrine NSCLC occurred in 2% of cases (95% CI, 2%–3%). The pooled median time from EGFR-TKI initiation to histological transformation was 19.39 months (95% CI, 16.18–22.60). After transformation, the pooled median progression-free survival (mPFS) was 3.93 months (95% CI, 3.14–4.72), and the pooled median overall survival was 10.69 months (95% CI, 8.34–13.04).

**Conclusions:**

Histological transformation occurs in a clinically relevant proportion of EGFR-TKI–resistant EGFR-mutant lung cancer, and is associated with poor post-transformation outcomes.

## Introduction

Lung cancer is one of the malignant tumors with the greatest public health burden worldwide, ranking first in both incidence and mortality among all types of tumors. According to the latest GLOBOCAN data from the International Agency for Research on Cancer (IARC), there were approximately 2.4 million new lung cancer cases globally in 2022, accounting for about 12.4% of all new cancer cases; lung cancer-related deaths were approximately 1.8 million, representing over 18% of all cancer deaths, remaining the leading cause of cancer-related mortality worldwide. [1]Among all lung cancer subtypes, non-small cell lung cancer (NSCLC) accounts for approximately 80%–85%, with lung adenocarcinoma being the most common histological type, representing 40%–50% of NSCLC cases. The incidence of lung adenocarcinoma is continuously increasing in Asian populations. A significant proportion of lung adenocarcinoma patients harbor oncogene alterations, especially mutations in the epidermal growth factor receptor (EGFR), which occur in 40%–60% of cases in Asian populations. This has made EGFR tyrosine kinase inhibitors (EGFR-TKIs) one of the standard first-line treatments for locally advanced and metastatic lung adenocarcinoma [2]. Although the use of EGFR-TKIs has significantly improved patient prognosis, the vast majority of patients eventually develop acquired resistance. Among these cases, 3–14% undergo histological transformation during the resistance process [3], such as transformation from adenocarcinoma to HGNEC (high-grade neuroendocrine carcinoma, including the transformation to small cell carcinoma or large cell neuroendocrine carcinoma) or NSCLC transformation (non-small cell lung cancer transformed to another phenotype of non-small cell lung cancer). Histological transformation involves complex mechanisms and often indicates a poorer prognosis. [4] The histological transformation of lung cancer has been one of the important factors contributing to TKI resistance. However, the description and mechanistic understanding of histological transformation after TKIs resistance in lung cancer remain insufficient. Numerous studies have shown that incidence of histological transformation after TKIs resistance ranges from 3% to 14%. However, this range is quite broad, and there are significant gaps in research regarding the transformation rates caused by different generations of TKIs. The treatment after histological transformation, survival time of patients after transformation, and the time to transformation are also unclear. Therefore, a systematic evaluation of the rate, time to transformation, molecular changes, and prognostic information of histological transformation after EGFR-TKIs resistance is of great value for guiding clinical decisions.

## Method and materials

This study was registered in PROSPERO(CRD420251272816).

PICO: Patients: EGFR mutant lung cancer patients who develope resistance to EGFR-TKIs. Intervention: EGFR-TKI; Chemotherapy, Immune therapy or TKI for patients who underwent histological transformation. Comparison: This study is a single-arm meta-analysis. Primary outcome: frequency of histological transformation; Secondary outcomes: time to transformation, post-transformation PFS/OS, T790M loss.

### Search strategy

We systematically searched PubMed, Embase and Web of Science for relevant studies published from inception to November 2025. We conducted a search on PubMed and Embase up to November 18th using the following method: ((“drug resistance, neoplasm“[MeSH Terms] OR “treatment resistance“[Title/Abstract] OR “acquired resistance“[Title/Abstract] OR “disease progression“[Title/Abstract] OR “treatment failure“[Title/Abstract] OR “osimertinib“[All Fields] OR “gefitinib“[All Fields] OR “erlotinib“[All Fields] OR “afatinib“[All Fields] OR “dacomitinib“[All Fields] OR “TKI“[Title/Abstract] OR “tyrosine kinase inhibitor“[Title/Abstract]) AND (“lung neoplasms“[MeSH Terms] OR “carcinoma, non small cell lung“[MeSH Terms] OR “small cell lung carcinoma“[MeSH Terms]) AND (“histolog* transform*“[Title/Abstract] OR “phenotyp* transform*“[Title/Abstract] OR “phenotypic switching“[Title/Abstract] OR “squamous transformation“[Title/Abstract] OR “small cell transformation“[Title/Abstract] OR “ne transformation“[Title/Abstract] OR “lineage plasticity“[Title/Abstract] OR “sclc transformation“[Title/Abstract] OR “nsclc transformation“[Title/Abstract] OR “histolog* evolution“[Title/Abstract])). In WoS, a search was conducted using the keywords “histological transformation” and “lung cancer.” After the search, two researchers independently performed duplicate removal, title and abstract screening, and finally the full-text screening. Review articles, cell lines and animal experiments, case reports, and non-continuous case series were directly excluded. Prospective and retrospective studies, as well as continuous case series, were included for further screening.

#### Inclusion criteria

(1) The enrolled patients were adults(> 18 years old) with EGFR mutations. (2) The literature clearly specified the inclusion and exclusion criteria for patient selection. All patients who underwent re-biopsy after developing resistance to TKI treatment. (3) Data on histological transformation, including the types of transformation, were clearly presented. All confirmed transformation cases were verified through re-biopsy after after developing resistance to TKI treatment. (4) There was no special screening applied, and the cohort was consecutive. (5) The resistance cohort should include no less than ten patients, or there should be no less than three patients exhibiting histological transformation in consecutive case series.

#### Exclusion criteria

(1) Case reports or non-consecutive case series. (2) Histological transformation occurring after lung cancer treatments other than EGFR tyrosine kinase inhibitors (TKIs) during the observation period. (3) Studies that only performed liquid biopsy, or where the literature did not clearly state that tissue pathological biopsy was conducted to identify histological transformation. (4) Included patients with non-small cell lung cancer (NSCLC) who do not have EGFR mutations. (5) Insufficient data: key variables required for the meta-analysis could not be extracted.

### Data extraction

All data were independently extracted by two authors. The main information to be extracted included the number of enrolled patients, specifically those who underwent a second pathological biopsy after developing resistance to EGFR-TKIs; the medication regimens of the enrolled patients; the tissue types from pathological biopsies before and after TKIs resistance, as well as the counts of various transformation types; the transformation time, defined as the duration from the start of EGFR-TKIs treatment to the detection of histological transformation; progression-free survival time after transformation; overall survival time after transformation; and gene comparison before and after transformation, such as whether EGFR mutations were retained and whether the EGFR T790M mutation was retained.

Extraction of continuous variables such as time to transformation, post-transformation progression-free survival (PFS), and post-transformation overall survival (OS): Some studies directly provided the relevant information, we thus can directly extract these data. For studies with clearly presented Kaplan-Meier (KM) curves, two authors manually reconstructed individual patient survival times by extracting event times and censoring times directly from the published KM curves. Researchers used the online software “webplotDigitizer” to digitize the time-event coordinates. Each step down in the survival curve was considered an event occurrence, and censoring information was accurately identified based on the censoring marks on the original KM curve. Event times were recorded according to the x-axis scale of the original chart. Validation of the extracted data involved regenerating the KM curves and comparing them with the originally published figures to verify the accuracy of the reconstructed individual survival data. The reconstructed data were reanalyzed for survival using SPSS, and the results were compared with those reported in the original articles. Ultimately, only data with minimal discrepancies were included in the final analysis.

### Quality assessment

The methodological quality of the included non-randomized controlled trials was assessed using the Joanna Briggs Institute (JBI) Critical Appraisal Checklist for Case Series. [5] This validation tool includes 10 items designed to assess key areas of methodological rigor. According to the information provided in the publication, each criterion is rated as “Yes” (criterion met), “No” (criterion not met), “Unclear,” or “Not applicable.”

Two independent reviewers conducted the assessments, and any disagreements were resolved through consensus with a third reviewer. The results of the quality assessments are summarized in tabular form. It is important to note that studies were not excluded based on quality scores; rather, the assessment results were taken into account during data synthesis and interpretation of the findings.

### Statistical analyses

Due to objective limitations, this study could only employ a single-arm meta-analysis approach to quantitatively combine and analyze the reported rates of histological transformation, time to histological transformation, progression-free survival after transformation, and overall survival from previous studies. All statistical analyses were performed using R software (version 4.3.3), IBM SPSS Statistics 20, or Stata 17. The meta-analysis was primarily conducted using the R package meta (version 8.2.1). For survival analysis of individual patient data (IPD), SPSS 20 and the R package survminer (version 0.5.1) were mainly used.

A single-arm meta-analysis was conducted to estimate the pooled proportion of histological transformation across studies. Proportions were logit-transformed to stabilize variances prior to pooling. Summary estimates were calculated using an inverse-variance weighted random-effects model, accounting for between-study heterogeneity. The between-study variance (τ²) was estimated using the restricted maximum-likelihood (REML) method. Hartung–Knapp adjustment was applied to derive 95% confidence intervals for pooled estimates. Subgroup differences in pooled proportions were assessed using a Q test for heterogeneity between subgroups.

For continuous time variables, histological transformation time, the median overall survival (mOS) after transformation is directly extracted from tables or conclusions in the original literature. If the data is not directly provided, patient-level individual participant data (IPD) is extracted from Kaplan-Meier (KM) curves using the online software WebPlotDigitizer. After extraction, the KM curves are redrawn and overlaid with the originally published KM curves to assess the goodness of fit. Meanwhile, the reconstructed IPD is analyzed for survival using SPSS to obtain the median values and confidence intervals for histological transformation time and post-transformation mOS required for this study. Finally, a random-effects model weighted by the inverse variance is used to pool the medians and their 95% confidence intervals for time to histological transformation and mOS after transformation. Similarly, inter-study heterogeneity is assessed using the I² statistic, and when significant heterogeneity is present, the random-effects model is preferred.

The IPD mPFS data is also extracted by the method above. After extraction of mPFS, a pool KM survival analysis is performed to compare the mPFS of patients underwent HGNEC transformation with different regimens. Random-effects model is performed at the same time.

The meta-analysis results are visualized using forest plots, reporting the pooled effect sizes, 95% confidence intervals, and heterogeneity metrics (I² and τ²). Differences between KM groups are assessed using the log-rank test. All statistical tests are two-sided, with a significance level set at *P* < 0.05.

### Publication bias and sensitivity analysis

Publication bias was assessed when at least 10 studies were available for a given outcome. Funnel plots were visually inspected for asymmetry, and statistical evaluation of small-study effects was performed using Egger’s regression test and Begg’s rank correlation test.

Sensitivity analyses were conducted to assess the robustness of the pooled estimates. A leave-one-out analysis was performed by sequentially excluding each study and recalculating the pooled effect size to evaluate the influence of individual studies on the overall results. In addition, sensitivity analyses were performed by excluding studies with small sample sizes or those with a higher risk of bias, when applicable. The stability of pooled estimates across these analyses was used to assess the reliability of the findings.

Sensitivity analyses and assessments of publication bias were performed for all primary outcomes and are reported in detail in the Supplementary Materials. (Figure S1-S8)

## Results

### Study selection

**Fig. 1:**
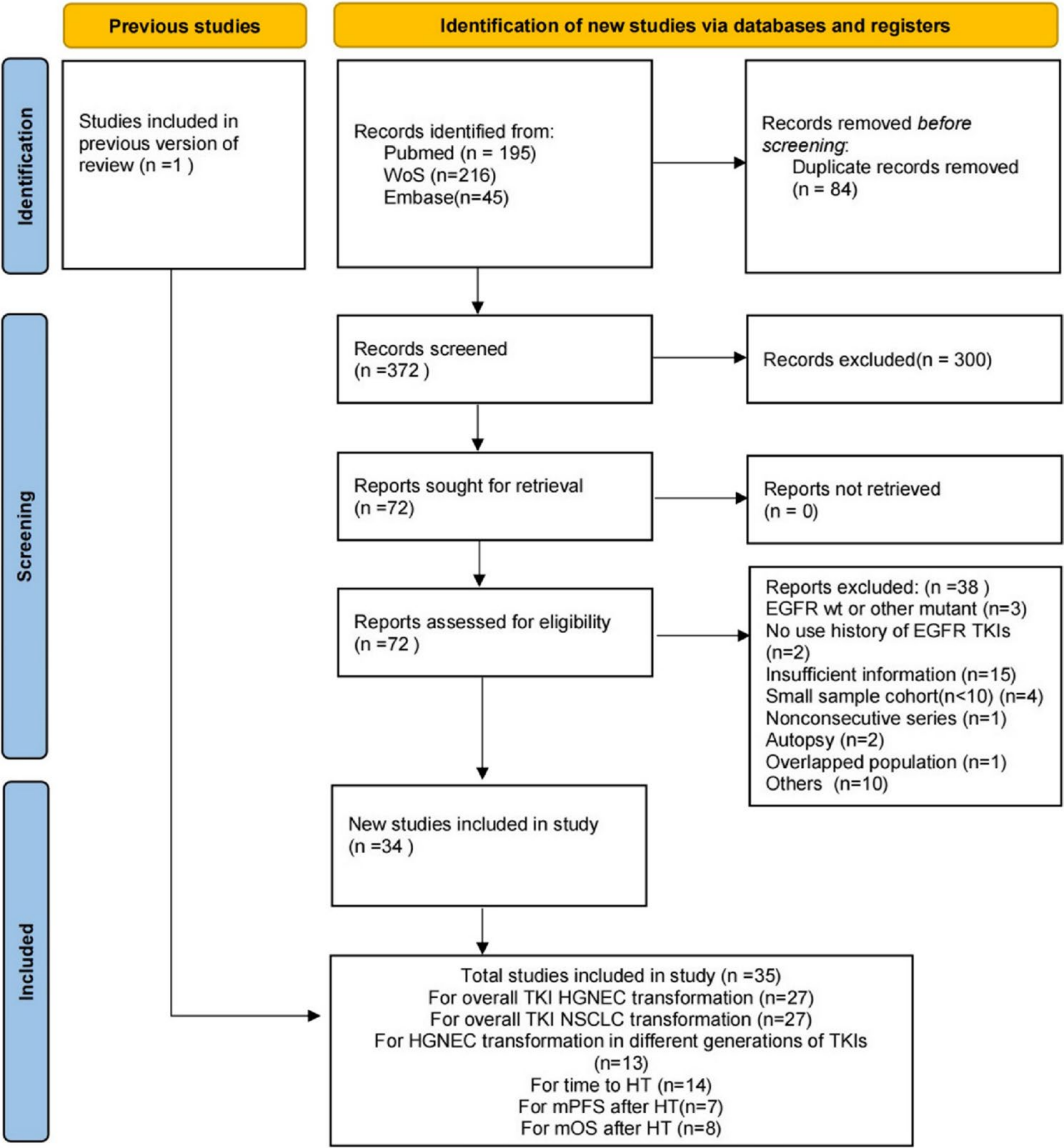
PRISMA 2020 Flow chart of study selection

A keyword search on PubMed yielded 195 results. A search on WoS returned 216 results. A search on Embase returned 45 results In total, there were 456 studies; after removing 84 duplicates, the titles and abstracts of the remaining 372 studies were screened. Full-text screening was applied to 72 studies, of which 38 were excluded. Additionally, one eligible study was included from the references of previous reviews [6]. Finally, 35 studies were included in the single-arm meta-analysis [3, 6–39]. Among them, 27 studies were used to calculate the proportion of EGFR-TKI-resistant patients who transformed into HGNEC, and 27 studies were used to calculate the proportion of EGFR-TKI-resistant patients who transformed into NSCLC [3, 6–14, 16–20, 22, 26–29, 32–36, 38, 39]. 14 articles were used for time to histological transformation analysis [3, 11, 13, 15, 23–25, 27, 28, 30–32, 38, 39]; 7 studies were used for the analysis of median progression-free survival time after transformation [15, 23–25, 30, 31, 37]; Eight studies were used for the analysis of median survival time after transformation [15, 23, 24, 27, 30, 35, 37, 38]. The specific process is shown in Fig. 1.

### Study characteristics

Overall, the included non-randomized studies demonstrated methodological quality ranging from moderate to high. Most studies defined inclusion criteria, employed reliable diagnostic methods, and consistently reported outcomes and follow-up data. For literature lacking specific relevant data, Kaplan-Meier (KM) curves were provided as substitutes. All studies explicitly stated the criteria for patient inclusion; all enrolled patients had EGFR mutations, had developed resistance to EGFR-TKIs, and had undergone two biopsies. Appropriate statistical methods were used in all studies. Clinical outcomes and follow-up results were also consistently reported. Cohorts that were not consecutively enrolled were directly excluded. Finally, the quality of the included studies was assessed using the JBI case series tool. Study characteristics and JBI assessment results are shown in Tables 1 and 2.

**Table 1:**
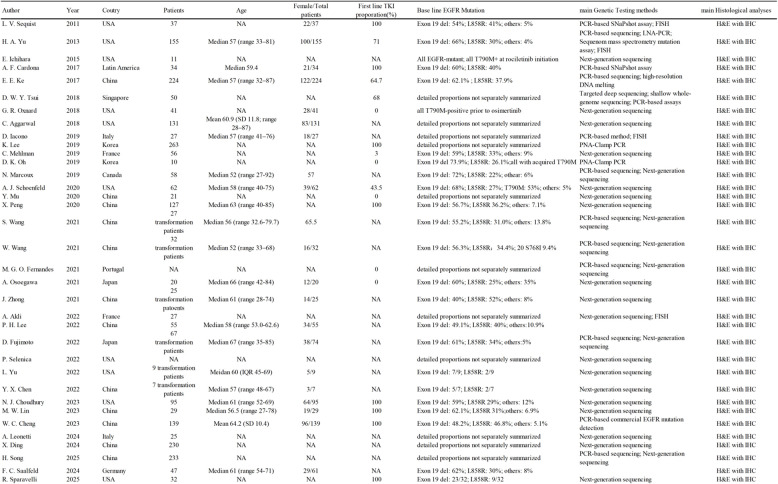
Study characteristics of all study included for single-arm meta-analysis

*NA* not found

**Table 2:**
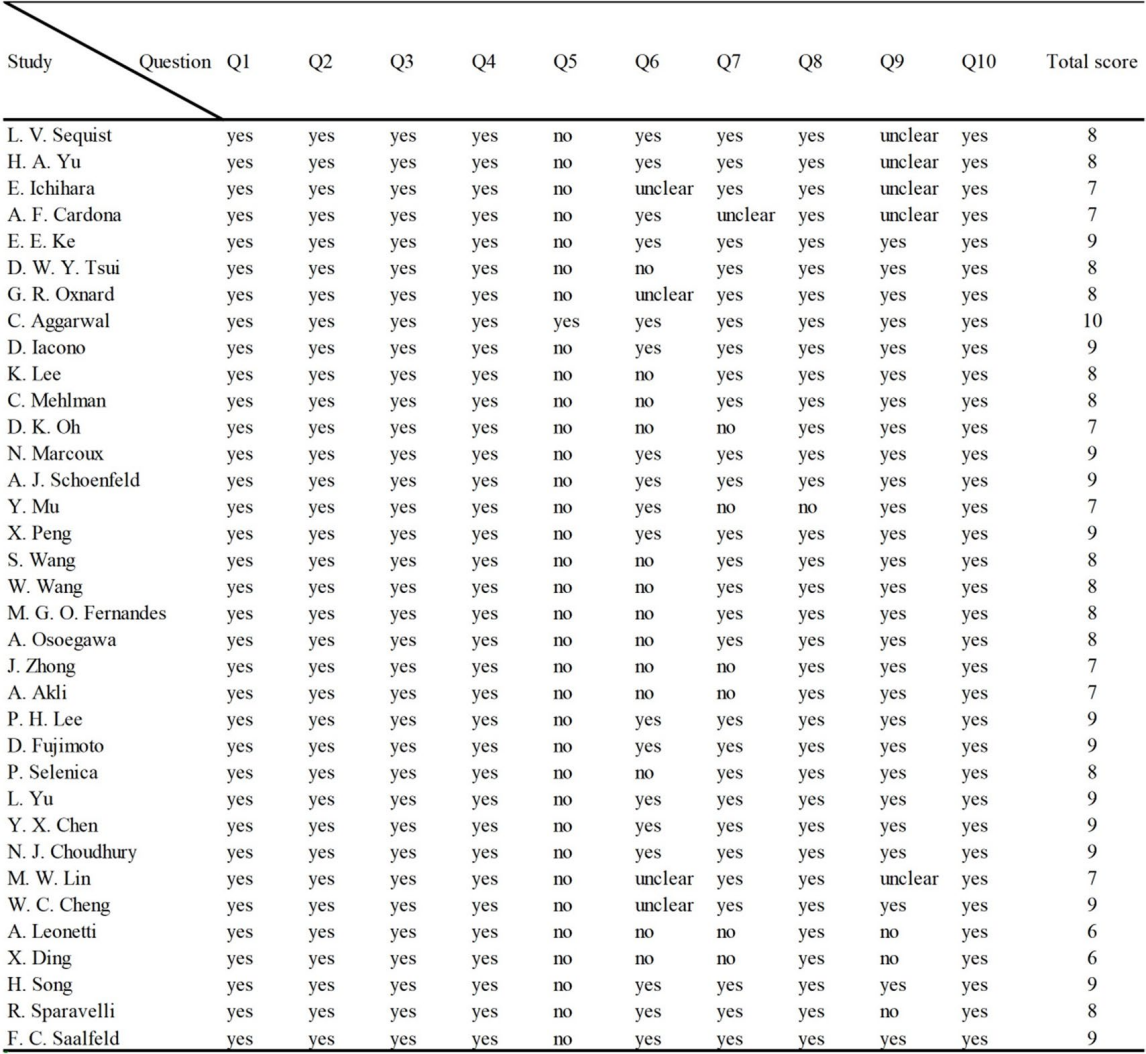
JBI checklist for case series

Q1: Were there clear criteria for inclusion in the case series? Q2: Was the condition measured in a standard, reliable way for all participants included in the case series? Q3: Were valid methods used for identification of the condition for all participants included in the case series? Q4: Did the case series have consecutive inclusion of participants? Q5: Did the case series have complete inclusion of participants? Q6: Was there clear reporting of the demographics of the participants in the study? Q7: Was there clear reporting of clinical information of the participants? Q8: Were the outcomes or follow up results of cases clearly reported? Q9: Was there clear reporting of the presenting site(s)/clinic(s) demographic information? Q10: Was statistical analysis appropriate?

Across included studies, biopsy procedures, immunohistochemical confirmation, and molecular testing platforms were variably reported. Some studies provided detailed descriptions of re-biopsy procedures, specimen types, neuroendocrine marker panels, and sequencing platforms, whereas others reported only the final pathological or genomic findings without sufficient methodological detail. This inconsistency may have contributed to between-study heterogeneity and limited cross-study comparability.

### Main result

#### Proportion of histological transformation

**Fig. 2:**
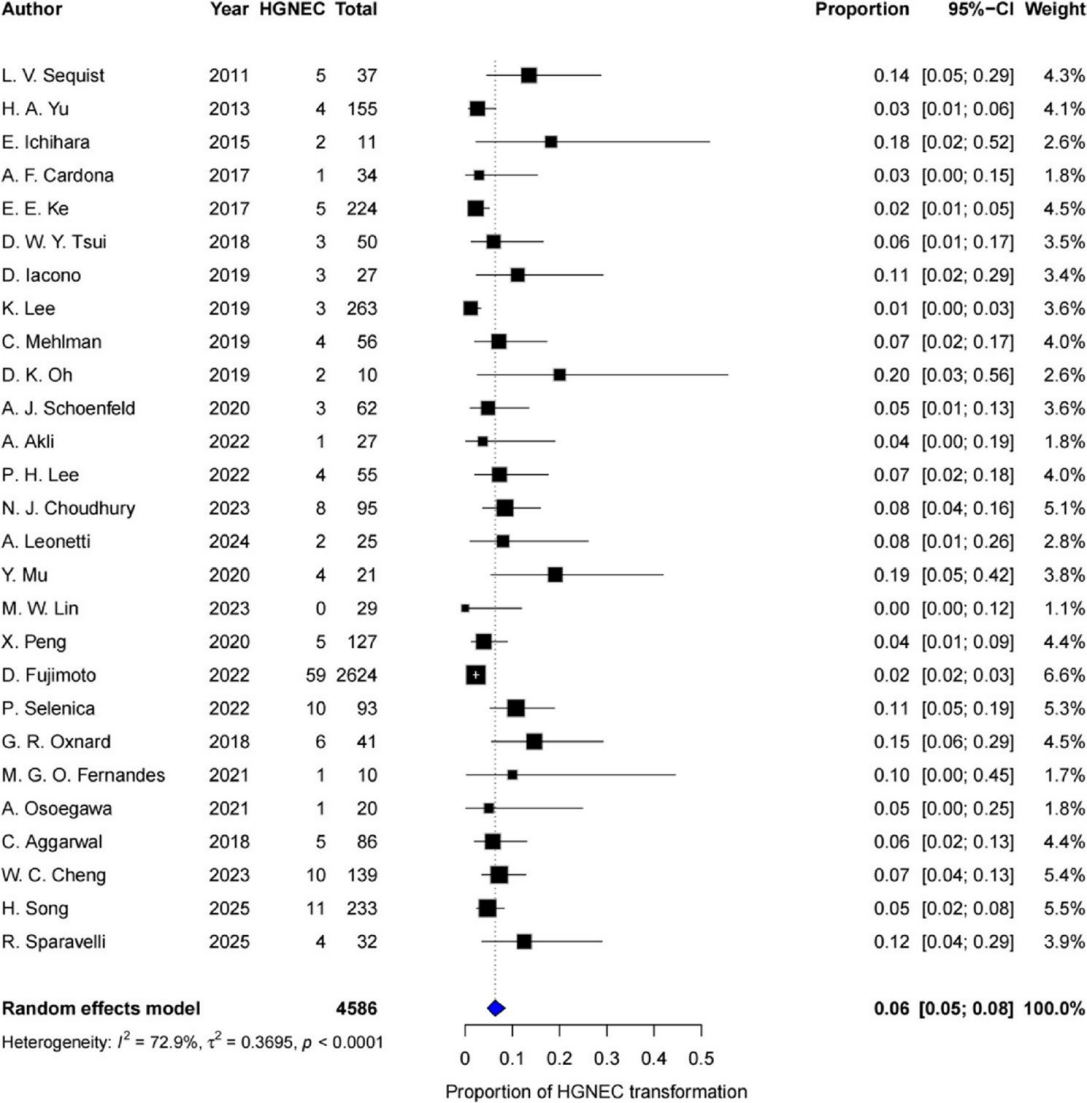
Proportion of HGNEC transformation. Forest plot showing the pooled proportion of high-grade neuroendocrine carcinoma (HGNEC) transformation among patients with EGFR-TKI–resistant non–small cell lung cancer (NSCLC)

Among all the included studies, 27 studies collectively enrolled a total of 4586 patients with EGFR-TKIs-resistant NSCLC. HGNEC transformation occurred in 166 cases. The proportion of patients who developed high-grade neuroendocrine carcinoma (HGNEC) transformation was 6% (95% CI, 5–8%). In the pooled population, most of the transformations were to small cell lung cancer (SCLC) and only 15 cases transformed to large cell neuroendocrine carcinoma (LCNEC). (Fig. 2)

**Fig. 3:**
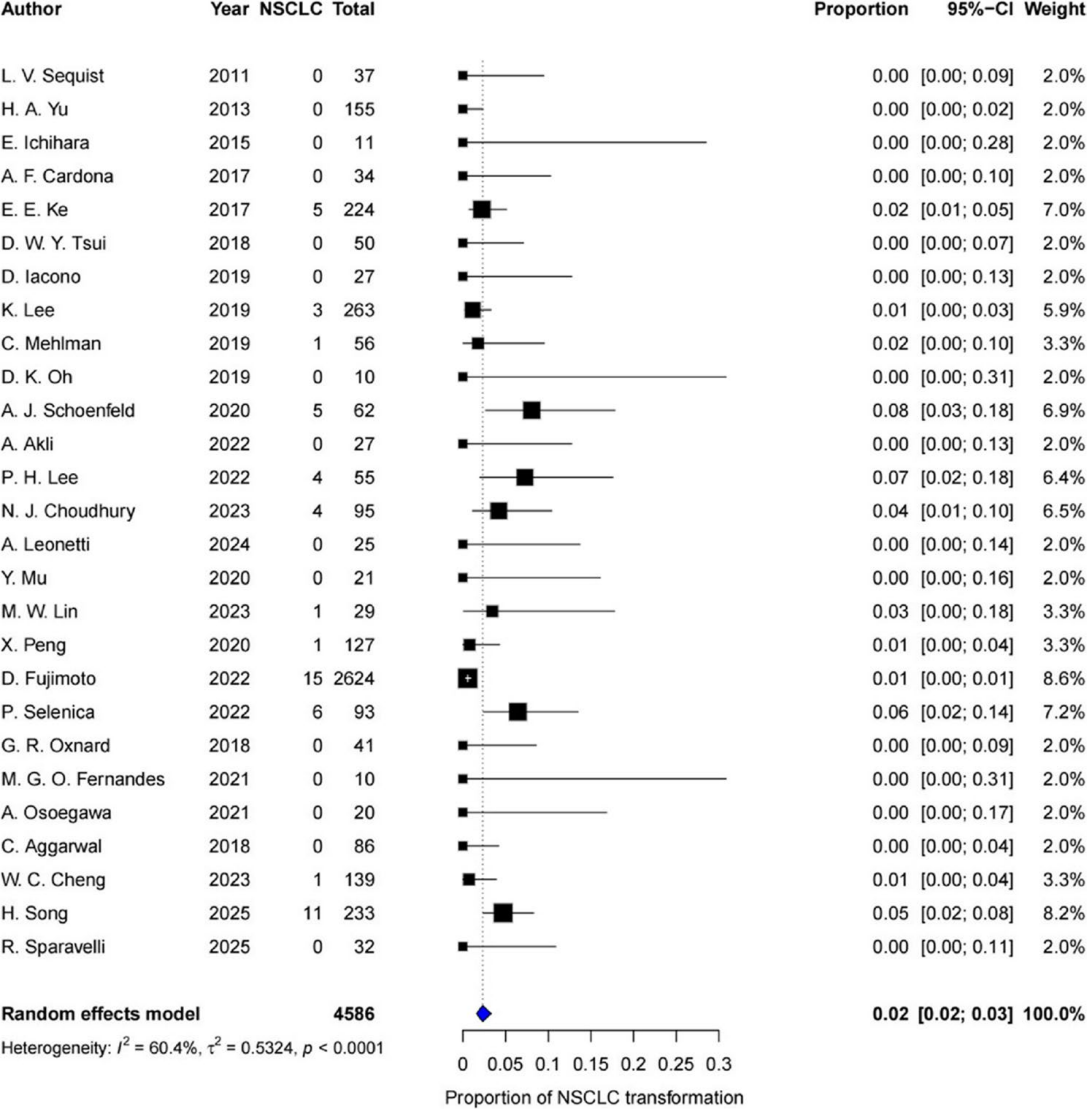
Proportion of NSCLC transformation. Forest plot showing the pooled proportion of non–small cell lung cancer (NSCLC) transformation among patients with EGFR-TKI–resistant non–small cell lung cancer (NSCLC)

In the meta-analysis of NSCLC histological transformation—which predominantly involved transformation from adenocarcinoma to squamous cell carcinoma, with a smaller proportion of cases transforming from adenocarcinoma to adenosquamous carcinoma and from squamous cell carcinoma to adenocarcinoma—a total of 27 studies comprising 4586 EGFR-TKI–resistant NSCLC patients were included. The pooled proportion of NSCLC histological transformation was 2% (95% CI, 2%–3%).(Fig. 3).

In clinical practice, later-line osimertinib exposure after T790M-mediated resistance appears to represent a clinically distinct setting from frontline third-generation EGFR-TKI treatment and likely reflects the combined effects of prior EGFR-TKI selection pressure, resistance-associated clonal evolution, and subsequent osimertinib exposure. This difference in treatment context may also contribute to between-study heterogeneity.

To further explore the potential impact of treatment-line heterogeneity in HGNEC transformation, we conducted additional subgroup analyses according to treatment line and EGFR-TKI generation. The studies were stratified into three categories: first-line first-/second-generation EGFR-TKI therapy, first-line third-generation EGFR-TKI therapy, and later-line third-generation EGFR-TKI therapy. Notably, in the later-line third-generation EGFR-TKI therapy group, only four cases from one study [20] received later-line osimertinib without documented T790M acquisition.

**Fig. 4:**
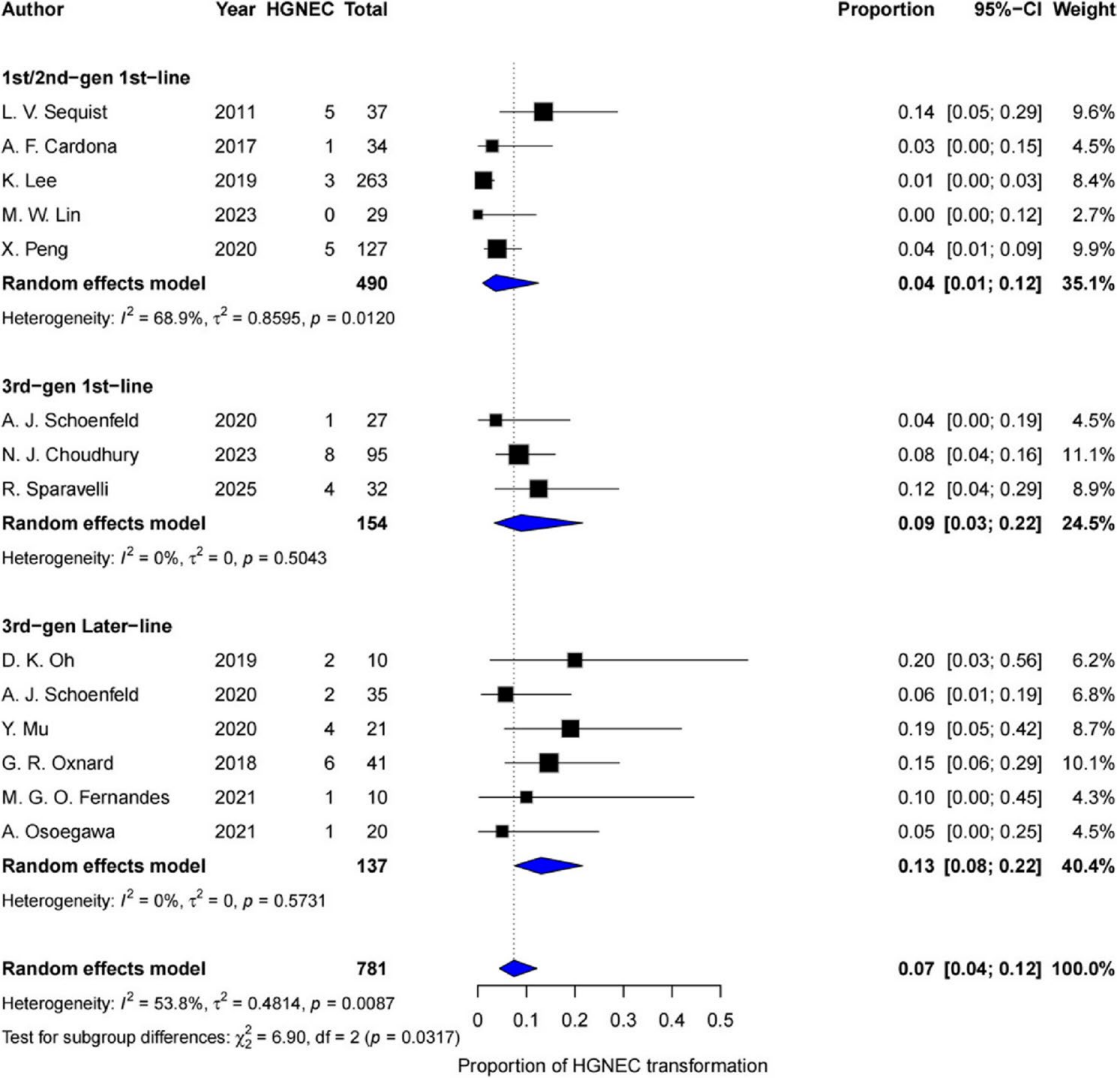
Proportion of HGNEC transformation (group by different lines and TKI generation). Forest plot showing the pooled proportion of HGNEC transformation among patients with EGFR-TKI–resistant non–small cell lung cancer (NSCLC) in different therapy lines and TKI generation

In Fig. 4, the overall test for subgroup differences was statistically significant (chi-square = 6.90, df = 2, *p* = 0.0317). In pairwise meta-regression using the first-line 1/2-gen EGFR-TKIs subgroup as the reference, the later-line third-generation subgroup showed a nominally significant difference (*p* = 0.0311), whereas the first-line third-generation subgroup did not (*p* = 0.1646). However, after Bonferroni correction for multiple pairwise comparisons, these differences were no longer statistically significant. In exploratory pairwise meta-regression using the first-line third-generation EGFR-TKI subgroup as the reference, neither the first-line 1/2-gen EGFR-TKIs subgroup nor the later-line third-generation subgroup showed a statistically significant difference (*p* = 0.1646 and *p* = 0.4864, respectively). These findings suggest that, although the overall subgroup test was significant, the pairwise differences involving frontline third-generation EGFR-TKI exposure were not statistically significant and should therefore be interpreted cautiously. Thus, the subgroup findings should not be overinterpreted.

Accordingly, frontline and T790M-mediated later-line osimertinib exposure should not be interpreted as equivalent clinical contexts, and the corresponding subgroup findings are best regarded as exploratory.

#### Median time to histological transformation

Due to the limitation of the study number, we only analyse HGNEC transformation subgroup.

**Fig. 5:**
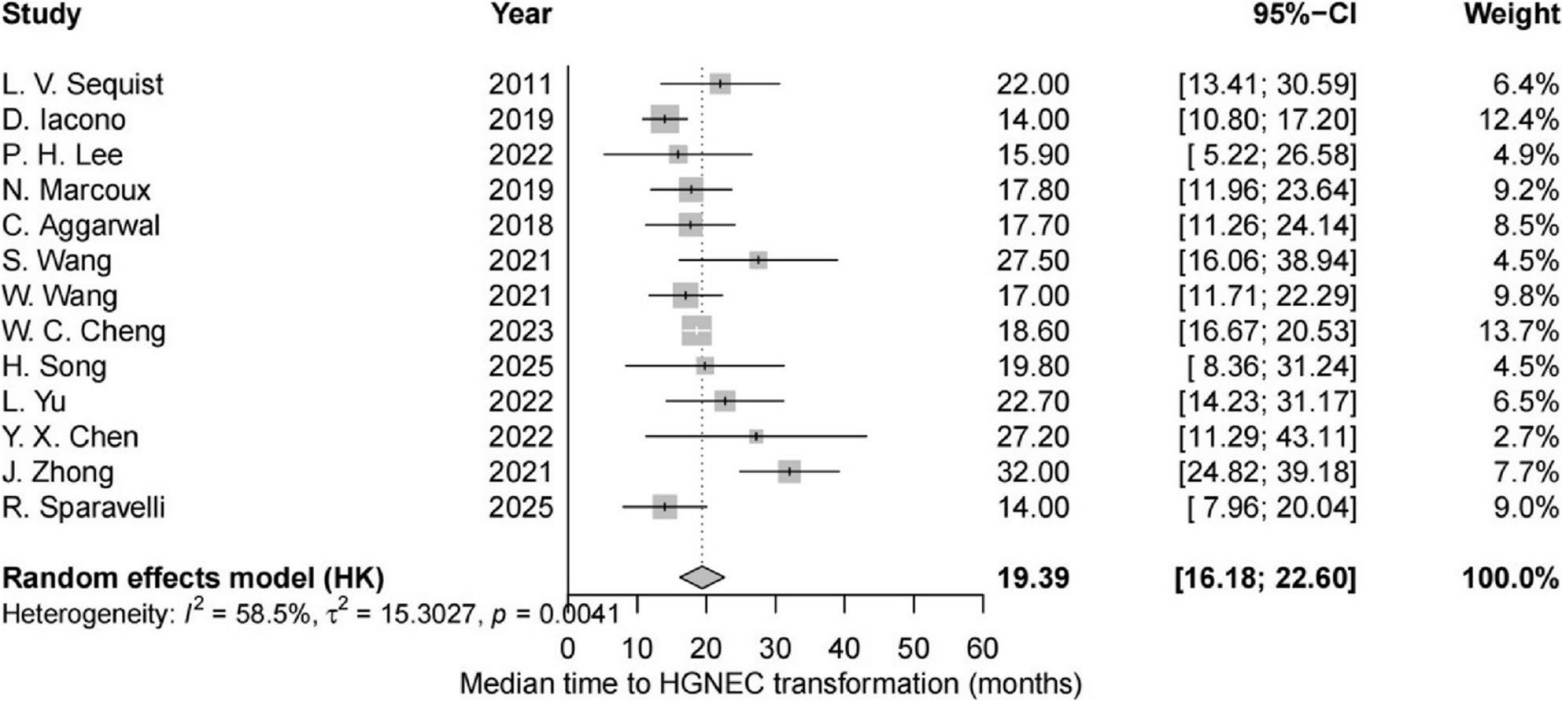
Time to HGNEC transformation. Forest plot showing the pooled median time from initiation of epidermal growth factor receptor tyrosine kinase inhibitor (EGFR-TKI) therapy to HGNEC transformation in patients with EGFR-mutant non–small cell lung cancer (NSCLC)

This meta-analysis examines the median time to HGNEC transformation in lung cancer patients who developed EGFR-TKI-resistance. The pooled estimate reveals a median transformation time of 19.39 months (95% CI, 16.18–22.60), with substantial heterogeneity observed across studies (I²=58.5%, τ²= 15.3027, *p* = 0.0041). The results underscore variability in transformation timing, ranging from 14.00 months to 32.00 months, depending on the study and patient characteristics. (Fig. 5).

#### mPFS after histological transformation

**Fig. 6:**
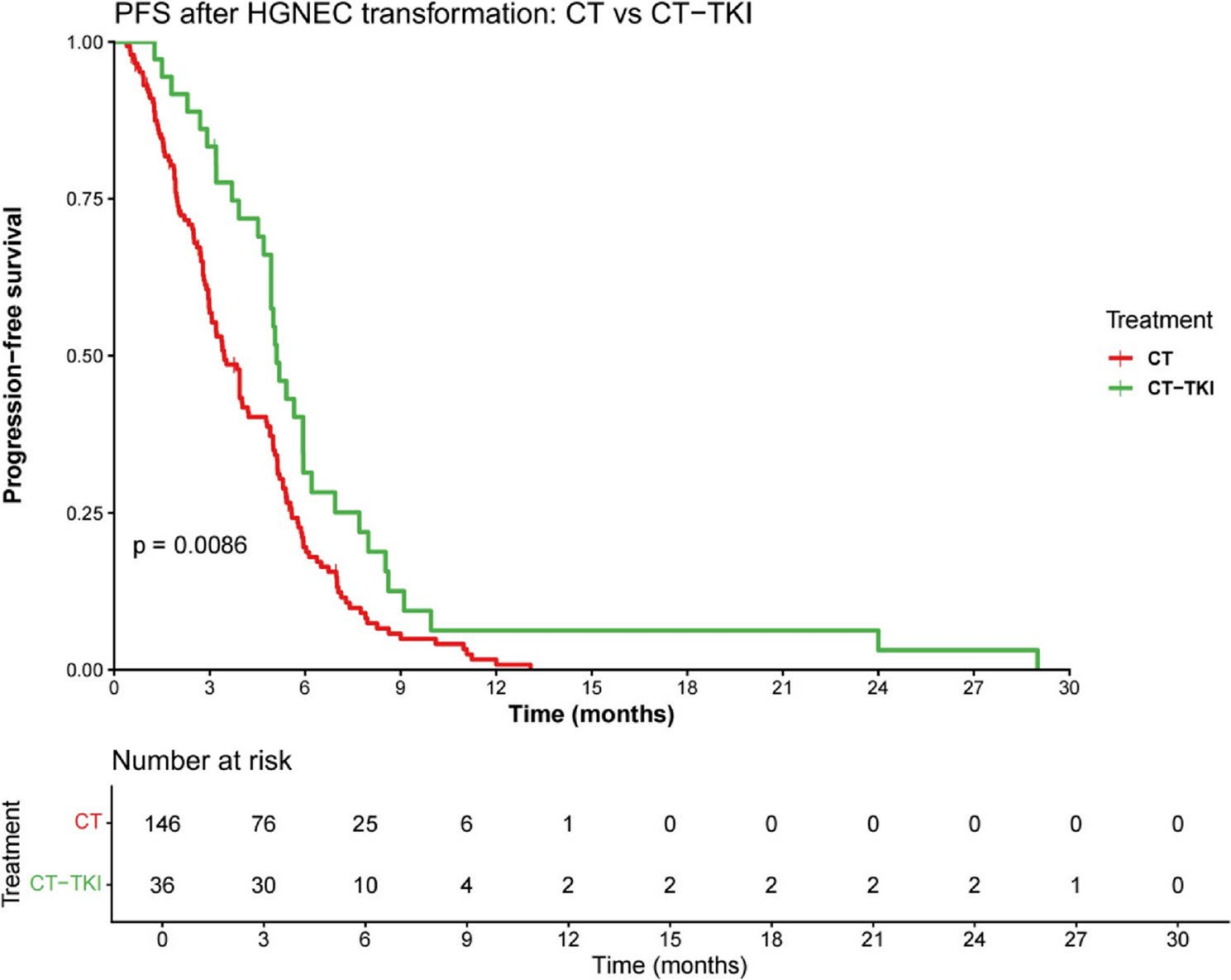
mPFS after HGNEC transformation (pool KM curve): Kaplan–Meier curves showing progression-free survival (PFS) after HGNEC transformation in patients with EGFR-mutant non–small cell lung cancer (NSCLC), stratified by post-transformation treatment regimen. CT vs. CT-TKI: Chemotherapy (CT) vs. chemotherapy plus EGFR-TKI (CT-TKI)

For the seven retrospective studies in which individual patient–level median progression-free survival (mPFS) data could be extracted, individual patient data (IPD) were reconstructed from the published Kaplan–Meier curves using the WebPlotDigitizer online software. The extracted IPD were cross-validated against the original published data, and all reconstructed datasets from the seven studies were successfully verified. Individual-level data from all studies were subsequently pooled, and survival analyses were performed with post-transformation treatment regimen as the stratification variable. The survival analysis demonstrated significant differences in mPFS among treatment groups. We compared the treatment outcomes of the two major therapeutic strategies reported in these studies and performed pairwise comparisons. (Fig. 6) Overall, combination therapy demonstrated superior efficacy compared with monotherapy. The mPFS time for chemotherapy alone was 3.46 months (95CI, 2.854–4.066). The mPFS time for EGFR-TKIs plus chemotherapy therapy was 5.10 months (95CI, 4.547–5.653).

**Fig. 7:**
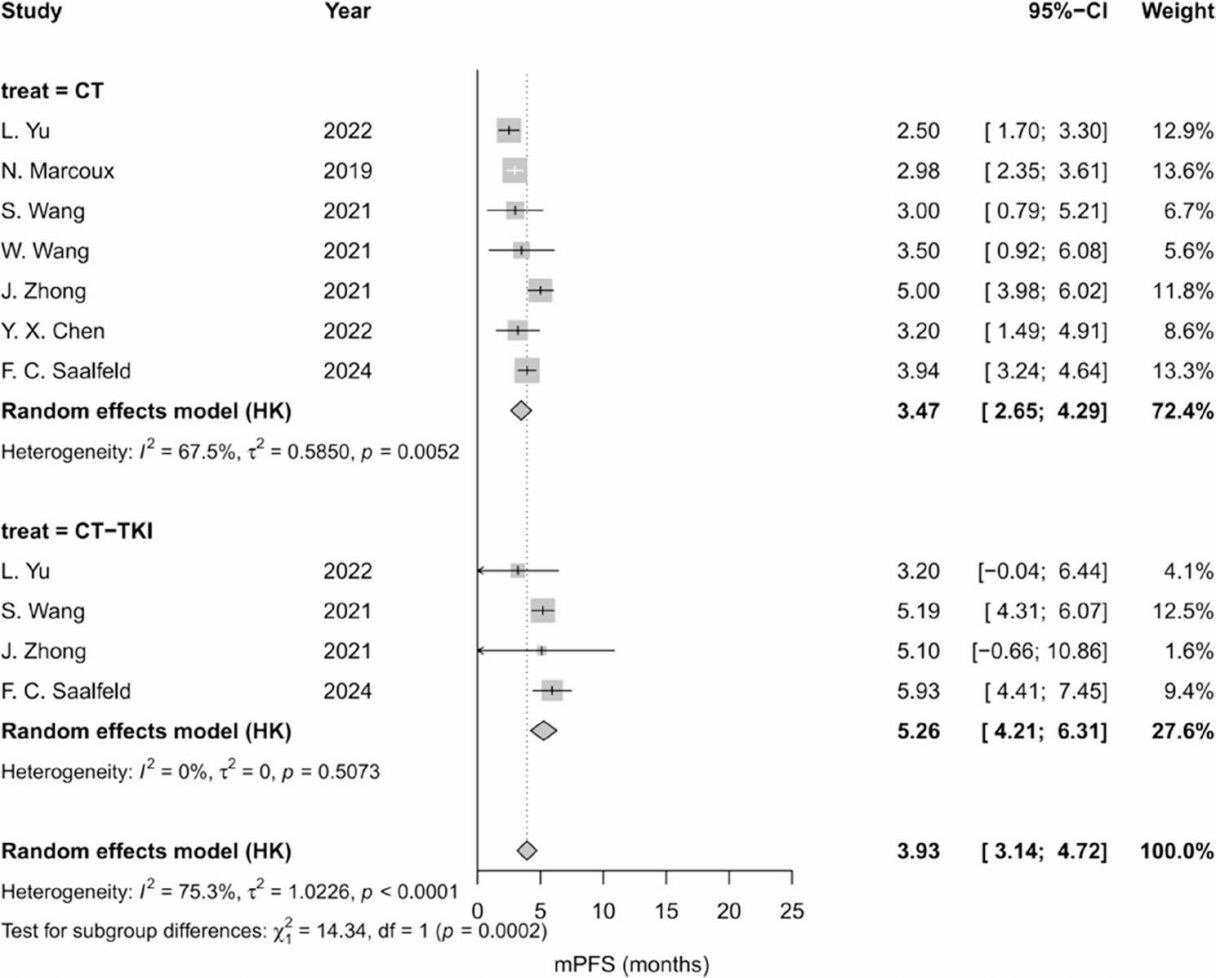
mPFS after histological transformation (Forest plot). Forest plot showing the pooled median progression-free survival (mPFS) after histological transformation in patients with EGFR-mutant non–small cell lung cancer (NSCLC), stratified by post-transformation treatment regimen

To further validate these findings, we additionally applied a random-effects meta-analysis to pool the mPFS estimates from the seven studies and conducted subgroup analyses according to treatment strategy. The overall pooled mPFS was 3.93 months (95% CI, 3.14–4.72; I²=75.3%). In subgroup analyses, the pooled mPFS was 5.26 months (95% CI, 4.21–6.31; I²=0%) for chemotherapy plus EGFR-TKIs, compared with 3.47 months (95% CI, 2.65–4.29; I²=67.5%) for chemotherapy alone. The difference between subgroups was statistically significant (chi-square = 14.34, *p* = 0.0002). Taken together, the random-effects pooled analysis yielded consistent results, indicating that among EGFR-TKI–resistant patients who undergo histological transformation, continuation of EGFR-TKIs in combination with chemotherapy is associated with a significantly longer progression-free survival compared with chemotherapy alone. (Figs 6 and 7).

#### mOS after Histological Transformation

**Fig. 8:**
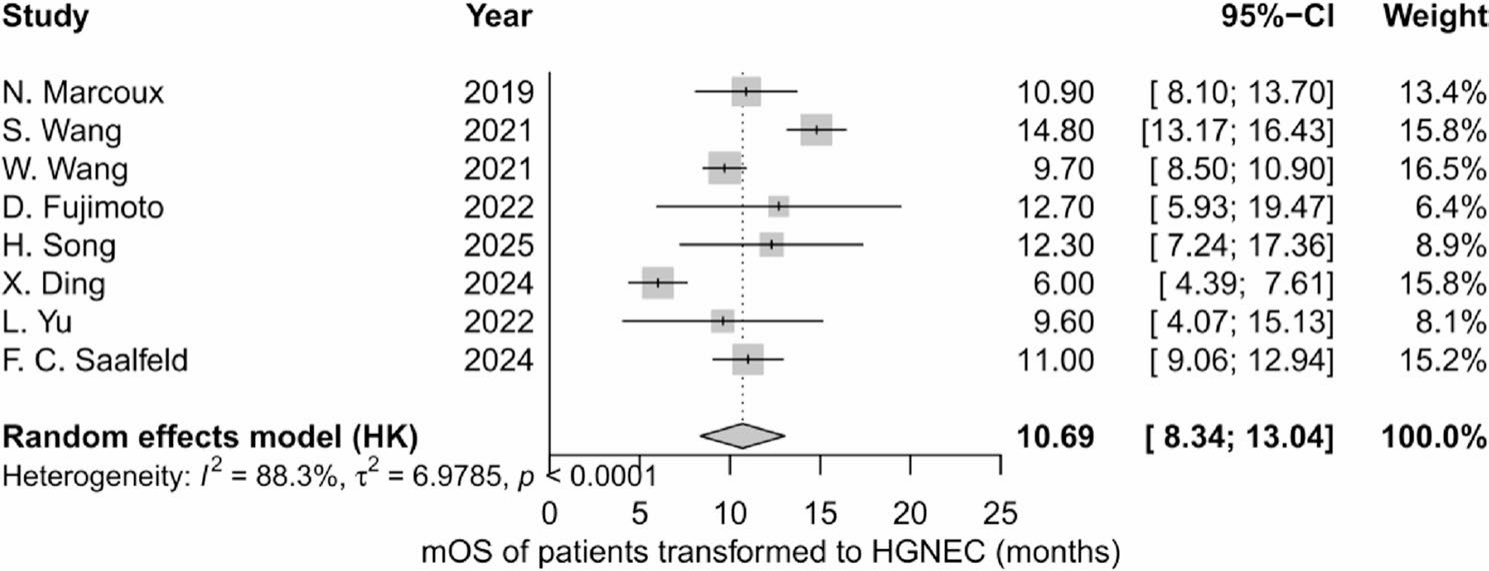
mOS after HGNEC transformation. Forest plot showing the pooled median overall survival (mOS) of patients with EGFR-mutant non–small cell lung cancer (NSCLC) after histological transformation to high-grade neuroendocrine carcinoma (HGNEC)

Among patients with high-grade neuroendocrine carcinoma arising from histological transformation—of whom more than 90% underwent transformation to small cell lung cancer (SCLC)—the median overall survival (mOS) was 10.69 months (95% CI, 8.34–13.04) (Fig. 8). Specifically, in a cohort of 678 patients with available clinical data, the median OS calculated from the date of SCLC diagnosis was 8.0 months (95% CI, 7.3–9.0) [40].

#### T790M-loss in the process of histological transformation

The T790M mutation plays a pivotal role in resistance to EGFR-TKIs. By increasing the affinity of the EGFR kinase domain for ATP, this mutation markedly diminishes the inhibitory activity of first- and second-generation EGFR-TKIs and represents the most common mechanism of acquired resistance. However, T790M mutation can also be lost during the 3rd -gen TKI therapy. The proportion of T790M loss among the overall population of patients with osimertinib resistance was only 50%. Mu, Y. et al. reported that among 49 patients who underwent molecular analysis, 24 patients retained the T790M mutation, whereas 25 patients exhibited loss of the T790M mutation [18]. By contrast, data extraction in the present review revealed that among patients who developed histological transformation, the proportion of T790M loss was as high as 80.7%. Notably, among the 16 studies that reported T790M status, 12 studies demonstrated a 100% loss rate of the T790M mutation (Table 3). However, It should be noted that the frequency of T790M loss observed in transformation cases may not be directly comparable with rates reported in unselected osimertinib-resistant populations. Transformation cases represent a biologically and clinically selected subgroup that underwent repeat biopsy and histological confirmation. Therefore, the higher proportion of T790M loss observed in these cases may partly reflect selection and sampling biases rather than a direct causal association. Therefore, this observation should be interpreted as a descriptive finding rather than evidence of a direct causal relationship.

**Table 3:** T790M-loss in the patients underwent histological transformation

Study	Year	Patients number	Histological Transformation	Transform to HGNEC	Transform to NSCLC	T790M-pre-tested	T790M-pre-pos	T790M-post-neg	T790M-loss(proportion)
E. Ichihara	2015	11	2	2	0	2	2	2	100.0%
R. Minari	2017	NR	5	4	0	5	5	5	100.0%
M. Offin	2019	34	7	7	0	7	1	1	100.0%
D. K. Oh	2019	10	2	2	0	2	2	2	100.0%
S. Park	2019	263	3	NR	3	1	1	1	100.0%
A. Leonetti	2024	25	2	2	0	1	1	1	100.0%
N. Marcoux	2019	NR	58	NR	NR	58	19	15	78.9%
Y. Mu	2020	21	4	4	0	4	1	1	100.0%
M. S. Hsieh	2019	NR	6	0	0	6	2	0	0.0%
S. Wang	2021	NR	29	29	0	18	11	7	63.6%
G. R. Oxnard	2018	41	6	6	0	6	6	6	100.0%
I. Mambetsariev	2022	NR	9	9	0	9	1	1	100.0%
M. G. O. Fernandes	2021	10	1	1	0	1	1	0	0.0%
A. Osoegawa	2021	20	1	1	0	1	1	1	100.0%
C. Aggarwal	2018	86	5	5	0	1	1	1	100.0%
Y. X. Chen	2022	1012	7	7	0	7	2	2	100.0%
							**total**		
						129	57	46	80.7%
NR: no records									

## Discussion

### Mechanism of histological transformation: clonal selection or lineage transformation

At present, two main hypotheses have been proposed to explain the mechanisms underlying histological transformation: the clonal selection model and the lineage transformation model [4].

Recent studies have provided compelling experimental support for the lineage transformation hypothesis. Using single-cell lineage tracing in genetically engineered mouse models, it has been demonstrated that, in the context of RB1 loss and EGFR extinction, MYC activation can drive the transformation of lung adenocarcinoma (LUAD) into small cell lung cancer (SCLC). Upon EGFR inhibition by EGFR-TKIs, EGFR-mutant LUAD cells enter a transient “stem-like bottleneck state” characterized by high proliferative (cycling) capacity. Cells in this intermediate state begin to express neuronal-associated genes, such as CREB and SOX9, and acquire tolerance to MYC-driven oncogenic stress. Importantly, this intermediate state appears to be a critical determinant of whether histological transformation ultimately occurs [41]. Additional genetic events, such as PTEN loss or PI3K pathway activation, which mitigate MYC-induced cytotoxicity, represent the final steps required to complete the transformation process [42]. Collectively, these experimental findings strongly support the concept that SCLC transformation represents a cell lineage transition, rather than the outgrowth of a pre-existing resistant clone.

From a genomic instability perspective, Tan et al. further expanded this mechanistic framework in their 2025 whole-exome sequencing study. By comparing EGFR-mutant LUAD, transformed SCLC, and de novo SCLC, the authors demonstrated that although transformed SCLC retains the original EGFR mutations from the adenocarcinoma precursor, it exhibits markedly increased genomic instability, including elevated levels of homologous recombination deficiency (HRD), loss of heterozygosity (LOH), telomeric allelic imbalance (TAI), and a significantly higher frequency of uniparental disomy (UPD) events. The authors proposed that UPD may function as a “second hit”, leading to biallelic inactivation of tumor suppressor genes and thereby facilitating progression toward the small cell phenotype. These findings provide structural genomic evidence supporting a lineage evolution model rather than simple clonal selection [43]. Consistent with these observations, integrative multi-omics analyses have shown that histological transformation of EGFR-mutant LUAD into SCLC or other high-grade neuroendocrine carcinomas is fundamentally driven by lineage plasticity, rather than by the acquisition of novel driver mutations. Quintanal-Villalonga et al. [44] demonstrated through comprehensive analyses of whole-exome sequencing, transcriptomics, DNA methylation, and proteomics that pre-transformation LUAD and post-transformation SCLC share highly similar mutational landscapes, tumor mutational burden, and ploidy profiles, indicating clear clonal relatedness. In contrast, the transformation process is accompanied by profound transcriptional reprogramming and epigenetic remodeling, highlighting that neuroendocrine transformation is primarily governed by non-mutational mechanisms [45]. Clinical observations further corroborate this model. Although RB1 alterations are strongly associated with transformed SCLC, transformation can also occur in EGFR-mutant SCLC patients harboring wild-type RB1, and in some cases, transformation proceeds even more rapidly [46]. This discrepancy suggests that RB1 gene status and RB protein functional loss may be uncoupled, potentially due to epigenetic regulation. Supporting this notion, Yang et al. demonstrated that EHMT2 enhances methylation of the SFRP1 promoter, leading to reduced SFRP1 expression, subsequent activation of the WNT/β-catenin pathway, and ultimately facilitating TKI-induced neuroendocrine transformation [45]. At the signaling pathway level, histological transformation is characterized by coordinated remodeling of multiple oncogenic networks, including PI3K–AKT, and WNT signaling pathways [47], alongside sustained suppression of NOTCH signaling [37]. Together, these converging molecular events provide a coherent mechanistic basis for histological transformation driven by lineage plasticity rather than clonal selection.

## Intratumoral heterogeneity at baseline

Although accumulating molecular evidence suggests that true lineage plasticity and clonal evolution play important roles in EGFR-TKI resistance–associated histological transformation, we should never neglect intratumoral heterogeneity. In some cases [48], tumors may contain minor neuroendocrine or mixed histological components at the time of initial diagnosis that are not captured by limited biopsy sampling. This sampling limitation is inherent to small biopsy specimens and represents a potential source of bias when interpreting transformation events.

According to the theory, histological transformation may represent either genuine lineage transformation [41] from adenocarcinoma to small cell carcinoma or the expansion of a pre-existing small cell component within combined histology. [49] In the latter scenario, small cell clones may already exist at baseline but become dominant under treatment pressure, leading to earlier detection of transformation. Therefore, the variability in time to HGNEC transformation observed across studies may partly be due to the intratumoral heterogeneity at baseline. Because most included studies did not clearly distinguish between these two scenarios, this factor could not be systematically analyzed in the present meta-analysis.

Future studies are needed to better distinguish truly transformed lung cancers from tumors that harbor mixed histological components from the outset. A clearer distinction between these entities would be important for estimating their incidence more accurately, comparing treatment responses across subgroups, and improving our understanding of the biological mechanisms underlying these different evolutionary patterns.

### Histological transformation in metastatic lesions

In this meta-analysis, histological transformation was defined based on tissue biopsy–confirmed pathological diagnosis. Consequently, cases in which transformation was diagnosed through cerebrospinal fluid (CSF) cytology, particularly in patients with central nervous system (CNS) metastases such as brain metastases or leptomeningeal disease, were not systematically captured.

However, in clinical practice, re-biopsy of intracranial lesions is often challenging or infeasible [50], and transformation to small-cell lung cancer may instead be identified through CSF cytological examination. Therefore, restricting inclusion to tissue-confirmed cases may have led to underrepresentation of transformation events occurring in the CNS.

Nevertheless, we adopted tissue-based confirmation to ensure diagnostic consistency and comparability across studies, as the definition of histological transformation traditionally requires pathological confirmation of tumor histology. Future studies incorporating liquid biopsy approaches, including CSF cytology and molecular profiling, may provide a more comprehensive assessment of transformation events in patients with CNS involvement.

Moreover, in this meta-analysis, the site of re-biopsy (primary tumor versus metastatic lesions) was not consistently reported across the included studies, which precluded subgroup analyses based on biopsy location. Future studies with more detailed reporting of biopsy sites may help clarify whether transformation patterns differ according to tumor location.

### Other limitations of this study

Several limitations of the present study should be acknowledged. First, this analysis was conducted as a single-arm meta-analysis, which inherently provides lower evidentiary strength compared with meta-analyses based on randomized controlled trials (RCTs) or network meta-analyses. [51] Most of the included studies were observational in nature, primarily retrospective cohorts, and systematically described the phenomenon of histological transformation in lung cancer, including its incidence, time to transformation, and post-transformation survival outcomes. To date, prospective clinical studies focusing on histological transformation remain scarce, and no RCTs specifically addressing this phenomenon have been conducted, which limits the level of causal inference that can be drawn.

Second, in the meta-analytic process, some included studies enrolled substantially larger patient populations than others. As a result, these large-sample studies contributed disproportionately high weights in certain analyses, potentially influencing the pooled estimates and reducing the robustness of the conclusions. This limitation cannot be fully resolved through statistical adjustment alone and underscores the need for additional high-quality, well-designed clinical studies to achieve more balanced evidence synthesis. In some studies, the estimated 95% confidence intervals for mPFS included negative values, which can be attributed to small sample sizes and the resulting large standard errors.

Thirdly, several sources of heterogeneity should also be considered when interpreting the results of this meta-analysis. First, the included studies differed in baseline patient characteristics, including tumor stage, age distribution, sex ratio, smoking status, and ethnic background. These factors may influence both treatment response and the likelihood of histological transformation. Second, the studies were conducted across different time periods, during which diagnostic approaches and therapeutic strategies for EGFR-mutant NSCLC evolved substantially. Third, variability in pathological assessment and molecular testing methods across studies may also contribute to differences in reported transformation rates and clinical outcomes. Taken together, these factors introduce unavoidable heterogeneity that may influence the pooled estimates and should be considered when interpreting the findings of this study.

Another limitation of this study is the relatively limited sample size in several subgroup analyses. Histological transformation following EGFR-TKI resistance remains a relatively uncommon event, and detailed subgroup data were not consistently reported across studies. As a result, some pooled estimates may be based on a limited number of studies or patients, which may reduce the statistical robustness of these findings. Therefore, the results of subgroup analyses should be interpreted with caution, and further large-scale studies are warranted to validate these observations. sensitivity analyses showed no single study significantly influenced the overall estimates.

In addition, histological transformations that were detected only after subsequent systemic therapies were not included in the analysis. This strategy was adopted to preserve a clear temporal relationship between EGFR-TKI exposure and transformation events. However, as transformation may sometimes be identified during re-biopsy performed in later treatment lines [52], the frequency of transformation reported in this study may be underestimated.

## Conclusion

Our findings suggest that: the median time to HGNEC transformation is 19.39 months; Chemotherapy combined with EGFR-TKIs rechallenging may be associated with improved PFS compared with chemotherapy alone for EGFR patients with histological transformation after EGFR-TKIs resistance, which indicates the vital importance of secondary biopsy and secondary gene testing. However, it requires prospective validation.

## Data Availability

No datasets were generated or analysed during the current study.

## References

[1] Bray F, et al. Global cancer statistics 2022: GLOBOCAN estimates of incidence and mortality worldwide for 36 cancers in 185 countries. CA Cancer J Clin. 2024;74:229–63. 10.3322/caac.21834. 38572751 10.3322/caac.21834

[2] Molina JR, Yang P, Cassivi SD, Schild SE, Adjei AA. Non-small cell lung cancer: epidemiology, risk factors, treatment, and survivorship. Mayo Clin Proc. 2008;83:584–94. 10.4065/83.5.584. 18452692 10.4065/83.5.584 PMC2718421

[3] Sequist LV, et al. Genotypic and histological evolution of lung cancers acquiring resistance to EGFR inhibitors. Sci Transl Med. 2011;3:75ra26. 10.1126/scitranslmed.3002003. 21430269 10.1126/scitranslmed.3002003 PMC3132801

[4] Liu S, Xu T, Cao X, Li H, Jin R. Histological transformation in lung cancer: Mechanisms, clinical characteristics, and therapeutic approaches. Biochim Biophys Acta Rev Cancer. 2025;1880:189413. 10.1016/j.bbcan.2025.189413. 40784437 10.1016/j.bbcan.2025.189413

[5] Munn Z, et al. Methodological quality of case series studies: an introduction to the JBI critical appraisal tool. JBI Evid Synthesis. 2020;18 :2127–33. 10.11124/JBISRIR-D-19-00099 10.11124/JBISRIR-D-19-00099 33038125

[6] Oxnard GR, et al. Assessment of Resistance Mechanisms and Clinical Implications in Patients With EGFR T790M-Positive Lung Cancer and Acquired Resistance to Osimertinib. JAMA Oncol. 2018;4:1527–34. 10.1001/jamaoncol.2018.2969. 30073261 10.1001/jamaoncol.2018.2969 PMC6240476

[7] Yu HA, et al. Analysis of tumor specimens at the time of acquired resistance to EGFR-TKI therapy in 155 patients with EGFR-mutant lung cancers. Clin Cancer Res. 2013;19:2240–7. 10.1158/1078-0432.Ccr-12-2246. 23470965 10.1158/1078-0432.CCR-12-2246 PMC3630270

[8] Ichihara E, Lovly CM. Shades of T790M: Intratumor Heterogeneity in EGFR-Mutant Lung Cancer. Cancer Discov. 2015;5:694–6. 10.1158/2159-8290.Cd-15-0616. 26152920 10.1158/2159-8290.CD-15-0616 PMC4499857

[9] Cardona AF, et al. Acquired Resistance to Erlotinib in EGFR Mutation-Positive Lung Adenocarcinoma among Hispanics (CLICaP). Target Oncol. 2017;12:513–23. 10.1007/s11523-017-0497-2. 28620690 10.1007/s11523-017-0497-2

[10] Ke EE, et al. A Higher Proportion of the EGFR T790M Mutation May Contribute to the Better Survival of Patients with Exon 19 Deletions Compared with Those with L858R. J Thorac Oncol. 2017;12:1368–75. 10.1016/j.jtho.2017.05.018. 28576746 10.1016/j.jtho.2017.05.018

[11] Aggarwal C, et al. Influence of TP53 Mutation on Survival in Patients With Advanced EGFR-Mutant Non-Small-Cell Lung Cancer. JCO PRECISION Oncol. 2018;2. 10.1200/PO.18.00107. 10.1200/PO.18.00107 PMC6372114 30766968

[12] Tsui DWY, et al. Dynamics of multiple resistance mechanisms in plasma DNA during EGFR-targeted therapies in non-small cell lung cancer. EMBO Mol Med. 2018;10. 10.15252/emmm.201707945. 10.15252/emmm.201707945 PMC5991591 29848757

[13] Iacono D, et al. Intrapatient Molecular and Histologic Heterogeneity After First-generation or Second-generation TKI Therapy of NSCLC Patients: Potential Clinical Impact on Subsequent third-generation TKI Treatment. Am J Clin Oncol. 2019;42:845–50. 10.1097/coc.0000000000000615. 31644442 10.1097/COC.0000000000000615

[14] Lee K, et al. Repeat biopsy procedures and T790M rates after afatinib, gefitinib, or erlotinib therapy in patients with lung cancer. Lung Cancer. 2019;130:87–92. 10.1016/j.lungcan.2019.01.012. 30885357 10.1016/j.lungcan.2019.01.012

[15] Marcoux N, et al. EGFR-Mutant Adenocarcinomas That Transform to Small-Cell Lung Cancer and Other Neuroendocrine Carcinomas: Clinical Outcomes. J Clin Oncol. 2019;37:278–85. 10.1200/jco.18.01585. 30550363 10.1200/JCO.18.01585 PMC7001776

[16] Mehlman C, et al. Resistance mechanisms to osimertinib in EGFR-mutated advanced non-small-cell lung cancer: A multicentric retrospective French study. Lung Cancer. 2019;137:149–56. 10.1016/j.lungcan.2019.09.019. 31600593 10.1016/j.lungcan.2019.09.019

[17] Oh DK, et al. Efficacy, safety, and resistance profile of osimertinib in T790M mutation-positive non-small cell lung cancer in real-world practice. PLoS ONE. 2019;14:e0210225. 10.1371/journal.pone.0210225. 30625213 10.1371/journal.pone.0210225 PMC6326493

[18] Mu Y, et al. Acquired resistance to osimertinib in patients with non-small-cell lung cancer: mechanisms and clinical outcomes. J Cancer Res Clin Oncol. 2020;146:2427–33. 10.1007/s00432-020-03239-1. 32385709 10.1007/s00432-020-03239-1 PMC7382655

[19] Peng X, et al. Clinical impact of uncommon epidermal growth factor receptor exon 19 insertion-deletion variants on epidermal growth factor receptor-tyrosine kinase inhibitor efficacy in non-small-cell lung cancer. Eur J Cancer. 2020;141:199–208. 10.1016/j.ejca.2020.10.005. 33171317 10.1016/j.ejca.2020.10.005

[20] Schoenfeld AJ, et al. Tumor Analyses Reveal Squamous Transformation and Off-Target Alterations As Early Resistance Mechanisms to First-line Osimertinib in EGFR-Mutant Lung Cancer. Clin Cancer Res. 2020;26:2654–63. 10.1158/1078-0432.Ccr-19-3563. 31911548 10.1158/1078-0432.CCR-19-3563 PMC7448565

[21] Fernandes MGO, et al. Resistance Profile of Osimertinib in Pre-treated Patients With EGFR T790M-Mutated Non-small Cell Lung Cancer. Front Oncol. 2021;11. 10.3389/fonc.2021.602924. 10.3389/fonc.2021.602924 PMC8136429 34026599

[22] Osoegawa A, et al. High Incidence of C797S Mutation in Patients With Long Treatment History of EGFR Tyrosine Kinase Inhibitors Including Osimertinib. JTO Clin Res Rep. 2021;2. 10.1016/j.jtocrr.2021.100191. 10.1016/j.jtocrr.2021.100191 PMC8474195 34590037

[23] Wang S, et al. Comprehensive analysis of treatment modes and clinical outcomes of small cell lung cancer transformed from epidermal growth factor receptor mutant lung adenocarcinoma. Thorac Cancer. 2021;12:2585–93. 10.1111/1759-7714.14144. 34490724 10.1111/1759-7714.14144 PMC8487822

[24] Wang W, et al. Genomic alterations and clinical outcomes in patients with lung adenocarcinoma with transformation to small cell lung cancer after treatment with EGFR tyrosine kinase inhibitors: A multicenter retrospective study. Lung Cancer. 2021;155:20–7. 10.1016/j.lungcan.2021.03.006. 33714778 10.1016/j.lungcan.2021.03.006

[25] Zhong J, et al. Evolution and genotypic characteristics of small cell lung cancer transformation in non-small cell lung carcinomas. J Natl CANCER Cent. 2021;1:153–. 10.1016/j.jncc.2021.11.001. 39036802 10.1016/j.jncc.2021.11.001 PMC11256618

[26] Akli A, et al. Histomolecular Resistance Mechanisms to First-Line Osimertinib in EGFR-Mutated Advanced Non-Small Cell Lung Cancer: A Multicentric Retrospective French Study. Target Oncol. 2022;17:675–82. 10.1007/s11523-022-00915-9. 36129569 10.1007/s11523-022-00915-9

[27] Fujimoto D, et al. Histologic transformation of epidermal growth factor receptor-mutated lung cancer. Eur J Cancer. 2022;166:41–50. 10.1016/j.ejca.2022.02.006. 35278824 10.1016/j.ejca.2022.02.006

[28] Lee PH, et al. Histological Transformation after Acquired Resistance to the Third-Generation EGFR-TKI in Patients with Advanced EGFR-Mutant Lung Adenocarcinoma. Med (Kaunas). 2022;58. 10.3390/medicina58070908. 10.3390/medicina58070908 PMC9323036 35888627

[29] Selenica P, et al. APOBEC mutagenesis, kataegis, chromothripsis in EGFR-mutant osimertinib-resistant lung adenocarcinomas. Ann Oncol. 2022;33:1284–95. 10.1016/j.annonc.2022.09.151. 36089134 10.1016/j.annonc.2022.09.151 PMC10360454

[30] Yu L, et al. Clinicopathologic and molecular characteristics of EGFR-mutant lung adenocarcinomas that transform to small-cell lung cancer after TKI therapy. TRANSLATIONAL LUNG CANCER Res. 2022;11:452–61. 10.21037/tlcr-21-665. 10.21037/tlcr-21-665 PMC8988081 35399568

[31] Chen YX, et al. Clinical and molecular profiling of EGFR-mutant lung adenocarcinomas transformation to small cell lung cancer during TKI treatment. Front Oncol. 2023;13. 10.3389/fonc.2023.1308313. 10.3389/fonc.2023.1308313 PMC10770233 38188289

[32] Cheng WC, et al. The Feasibility of Interventional Pulmonology Methods for Detecting the T790M Mutation after the First or Second-Generation EGFR-TKI Resistance of Non-Small Cell Lung Cancer. DIAGNOSTICS. 2023;13. 10.3390/diagnostics13010129. 10.3390/diagnostics13010129 PMC9819002 36611420

[33] Choudhury NJ, et al. Molecular Biomarkers of Disease Outcomes and Mechanisms of Acquired Resistance to First-Line Osimertinib in Advanced EGFR-Mutant Lung Cancers. J Thorac Oncol. 2023;18:463–75. 10.1016/j.jtho.2022.11.022. 36494075 10.1016/j.jtho.2022.11.022 PMC10249779

[34] Lin MW, et al. Salvage Surgery for Advanced Lung Adenocarcinoma After Epidermal Growth Factor Receptor Tyrosine Kinase Inhibitor Treatment. Ann Thorac Surg. 2023;116:111–9. 10.1016/j.athoracsur.2023.01.027. 36739067 10.1016/j.athoracsur.2023.01.027

[35] Ding X, et al. Transformation to small cell lung cancer is irrespective of EGFR and accelerated by SMAD4-mediated ASCL1 transcription independently of RB1 in non-small cell lung cancer. Cell Commun Signal. 2024;22:45. 10.1186/s12964-023-01260-8. 38233864 10.1186/s12964-023-01260-8 PMC10795321

[36] Leonetti A, et al. Resistance to osimertinib in advanced EGFR-mutated NSCLC: a prospective study of molecular genotyping on tissue and liquid biopsies. Br J Cancer. 2024;130:135–42. 10.1038/s41416-023-02475-9. 37938348 10.1038/s41416-023-02475-9 PMC10781773

[37] Saalfeld FC, et al. Small cell transformation in EGFR-mutated non-small cell lung cancer: DLL3 expression and efficacy of immune checkpoint inhibitors or tyrosine kinase inhibitors combined with chemotherapy. Eur J Cancer. 2024;213:115065. 10.1016/j.ejca.2024.115065. 39423775 10.1016/j.ejca.2024.115065

[38] Song H, Kim D, Jang SJ, Hwang HS, Song JS. Clinicopathologic features of histologic transformation in lung adenocarcinoma after treatment with epidermal growth factor receptor-tyrosine kinase inhibitors. Ann Diagn Pathol. 2025;77:152478. 10.1016/j.anndiagpath.2025.152478. 40215564 10.1016/j.anndiagpath.2025.152478

[39] Sparavelli R, et al. Prevalence of Histological Transformation in First-Line Osimertinib Non-Small Cell Lung Cancers: Case Series and Literature Review. Int J Mol Sci. 2025;26. 10.3390/ijms262110462. 10.3390/ijms262110462 PMC12609510 41226501

[40] Sivakumar S, et al. Integrative Analysis of a Large Real-World Cohort of Small Cell Lung Cancer Identifies Distinct Genetic Subtypes and Insights into Histologic Transformation. Cancer Discov. 2023;13:1572–91. 10.1158/2159-8290.Cd-22-0620. 37062002 10.1158/2159-8290.CD-22-0620 PMC10326603

[41] Gardner EE, et al. Lineage-specific intolerance to oncogenic drivers restricts histological transformation. Science. 2024;383:eadj1415. 10.1126/science.adj1415. 38330136 10.1126/science.adj1415 PMC11155264

[42] Zhang L, et al. PTEN Loss Expands the Histopathologic Diversity and Lineage Plasticity of Lung Cancers Initiated by Rb1/Trp53 Deletion. J Thorac Oncol. 2023;18:324–38. 10.1016/j.jtho.2022.11.019. 36473627 10.1016/j.jtho.2022.11.019 PMC9974779

[43] Tan JJ, et al. Whole Exome Sequencing Study Identifies Distinct Characteristics of Transformed Small Cell Lung Cancer With EGFR Mutation Compared to De Novo Small Cell and Primary Non-Small Cell Lung Cancers. Cancer Med. 2025;14. 10.1002/cam4.70838. 10.1002/cam4.70838 PMC11976457 40197849

[44] Rakhade S et al. Signatures of early plasticity in the histological transformation of lung cancer. J Clin Oncol. 2024;42:8042–8042. 10.1200/JCO.2024.42.16_suppl.8042

[45] Quintanal-Villalonga A, et al. Multiomic Analysis of Lung Tumors Defines Pathways Activated in Neuroendocrine Transformation. Cancer Discov. 2021;11:3028–47. 10.1158/2159-8290.Cd-20-1863. 34155000 10.1158/2159-8290.CD-20-1863 PMC9437746

[46] Huang J, et al. Genotyping of RB1 status identifies two distinct subtypes in EGFR-mutant lung cancers with SCLC transformation. Clin Transl Med. 2024;14:e1683. 10.1002/ctm2.1683. 38736106 10.1002/ctm2.1683 PMC11089080

[47] Yang C, et al. EHMT2-mediated transcriptional reprogramming drives neuroendocrine transformation in non-small cell lung cancer. Proc Natl Acad Sci U S A. 2024;121:e2317790121. 10.1073/pnas.2317790121. 38814866 10.1073/pnas.2317790121 PMC11161775

[48] Baldelli E, Mandarano M, Bellezza G, Petricoin EF, Pierobon M. Analysis of neuroendocrine clones in NSCLCs using an immuno-guided laser-capture microdissection-based approach. Cell Rep Methods. 2022;2:100271. 10.1016/j.crmeth.2022.100271. 36046628 10.1016/j.crmeth.2022.100271 PMC9421534

[49] Li R, Jiang L, Zhou X, Lu Y, Zhang Y. Pseudo-small cell transformation in EGFR-mutant adenocarcinoma. Lung Cancer. 2021;153:120–5. 10.1016/j.lungcan.2020.12.036. 33486417 10.1016/j.lungcan.2020.12.036

[50] Carapella CM, Gorgoglione N, Oppido PA. The role of surgical resection in patients with brain metastases. Curr Opin Oncol. 2018;30:390–5. 10.1097/cco.0000000000000484. 30142093 10.1097/CCO.0000000000000484

[51] Barker TH, et al. Conducting proportional meta-analysis in different types of systematic reviews: a guide for synthesisers of evidence. BMC Med Res Methodol. 2021;21:189. 10.1186/s12874-021-01381-z. 34544368 10.1186/s12874-021-01381-z PMC8451728

[52] Jin CB, Yang L. Histological transformation of non-small cell lung cancer: Clinical analysis of nine cases. WORLD J Clin CASES. 2021;9:4617–26. 10.12998/wjcc.v9.i18.4617. 34222428 10.12998/wjcc.v9.i18.4617 PMC8223818

